# Effect of vaginal estrogen supplementation for luteal phase support in the GnRH antagonist protocol on pregnancy outcomes for IVF/ICSI cycles: a randomized controlled trial

**DOI:** 10.1093/hropen/hoag042

**Published:** 2026-05-11

**Authors:** Xinyun Yang, Linling Zhu, Ruizhe Chen, Tongtong Lin, Fang Le, Qitao Zhan, Huijuan Gao, Fen Wang, Yimin Zhu, Hangying Lou

**Affiliations:** Department of Reproductive Endocrinology, Women’s Hospital, Zhejiang University School of Medicine, Hangzhou, Zhejiang, P.R. China; Department of Gynecology, Hangzhou Women’s Hospital, Hangzhou, Zhejiang, P.R. China; Department of Reproductive Endocrinology, Women’s Hospital, Zhejiang University School of Medicine, Hangzhou, Zhejiang, P.R. China; Department of Reproductive Endocrinology, Women’s Hospital, Zhejiang University School of Medicine, Hangzhou, Zhejiang, P.R. China; Department of Reproductive Endocrinology, Women’s Hospital, Zhejiang University School of Medicine, Hangzhou, Zhejiang, P.R. China; Department of Reproductive Endocrinology, Women’s Hospital, Zhejiang University School of Medicine, Hangzhou, Zhejiang, P.R. China; Department of Reproductive Endocrinology, Women’s Hospital, Zhejiang University School of Medicine, Hangzhou, Zhejiang, P.R. China; Department of Gynecology, Xiaoshan District Jingjiang Subdistrict Community Health Service Center, Hangzhou, Zhejiang, P.R. China; Department of Reproductive Endocrinology, Women’s Hospital, Zhejiang University School of Medicine, Hangzhou, Zhejiang, P.R. China; Department of Reproductive Endocrinology, Women’s Hospital, Zhejiang University School of Medicine, Hangzhou, Zhejiang, P.R. China

**Keywords:** vaginal estradiol addition, progesterone, luteal phase support, GnRH antagonist, RCT, IVF, ICSI, pregnancy outcome, clinical pregnancy rate, ongoing pregnancy rate

## Abstract

**STUDY QUESTION:**

Does adding vaginal estradiol to standard progesterone luteal phase support improve ongoing pregnancy rates in normal-responder IVF/ICSI patients undergoing GnRH antagonist cycles?

**SUMMARY ANSWER:**

The addition of vaginal estradiol to progesterone luteal support in GnRH antagonist cycles, compared to progesterone alone, enhances embryo implantation and clinical pregnancy rates in normal-responder IVF/ICSI patients, yet fails to significantly improve ongoing pregnancy or live birth outcomes.

**WHAT IS KNOWN ALREADY:**

The GnRH antagonist protocol for IVF, while advantageous in some aspects, may negatively impact estrogen levels and endometrial receptivity, potentially lowering fresh embryo transfer pregnancy rates compared to agonist protocols. The role of adding estrogen to standard progesterone luteal phase support in antagonist cycles remains controversial, with existing studies yielding conflicting results and a lack of robust randomized controlled trials (RCTs) specifically investigating vaginal estrogen administration.

**STUDY DESIGN, SIZE, DURATION:**

In this single-centre RCT, participants were randomized 1:1 on the ovulation trigger day to receive either progesterone alone (P group) or progesterone plus vaginal 17β-estradiol (E + P group) for luteal phase support, starting on Day 1 post-oocyte retrieval. The sample size calculation, based on detecting a clinically significant 13% difference in ongoing pregnancy rate (43% vs 30%; α = 0.05, power = 80%) with an anticipated 10% attrition, determined a target of 236 women per group. A total of 518 women (P group: n = 259; E + P group: n = 259) were enrolled between April 2019 and June 2022. Allocation used computerized randomization by an independent data manager. Blinding of participants and physicians was not feasible; however, statisticians were blinded until analysis completion. The analysis was performed using intention-to-treat (ITT) and per-protocol (PP), followed by a sensitivity analysis using multivariable logistic regression.

**PARTICIPANTS/MATERIALS, SETTING, METHODS:**

This single-centre trial enrolled 518 infertile women (20–39 years) undergoing fresh IVF/ICSI cycles as normal responders at a tertiary care centre. Participants were allocated to either the P group or E + P group. Luteal support initiated on Day 1 post-oocyte retrieval comprised: vaginal progesterone 90 mg/day + oral dydrogesterone 10 mg twice daily for both groups, with the E + P group additionally receiving vaginal 17β-estradiol 2 mg nightly. The primary outcome was ongoing pregnancy rate, with secondary outcomes including the rates of implantation, clinical pregnancy, live birth, and maternal complications.

**MAIN RESULTS AND THE ROLE OF CHANCE:**

The ITT analysis showed no significant differences in ongoing pregnancy rates of 46.33% (120/259) in the E + P group versus 39.00% (101/259) in the P group (RR 1.19, 95% CI: 0.97–1.45; *P* = 0.091), with the PP analysis yielding 48.78% (120/246) versus 41.39% (101/244) (RR 1.18, 95% CI: 0.97–1.44; *P* = 0.1). Implantation rates, in both the ITT and PP analyses, favoured the E + P group (39.66% vs 32.96%; RR 1.20, 95% CI: 1.01–1.43; *P* = 0.035), and clinical pregnancy rates were significantly higher in the E + P group both by ITT (53.28% vs 44.02%; RR 1.21, 95% CI: 1.01–1.44; *P* = 0.035) and PP analyses (56.10% vs 46.72%; RR 1.20, 95% CI: 1.01–1.43; *P* = 0.038). No significant differences were observed in live birth rates. Notably, intrahepatic cholestasis of pregnancy (ICP) occurred more frequently in the E + P group (5.17% vs 0%, *P* = 0.032), however, this difference was no longer statistically significant after multivariable adjustment (Adjusted *P* = 0.996).

**LIMITATIONS, REASONS FOR CAUTION:**

The increase in ICP in the estradiol group is a major safety concern requiring cautious interpretation, despite the improvement in some intermediate outcomes, such as implantation and clinical pregnancy. The lack of significant benefit on the primary outcome, ongoing pregnancy, and live birth limits the clinical utility.

**WIDER IMPLICATIONS OF THE FINDINGS:**

The addition of vaginal luteal estradiol supplementation in GnRH antagonist cycles improves embryo implantation and clinical pregnancy rates, yet fails to demonstrate significant benefits in ongoing pregnancy or live birth outcomes for IVF/ICSI patients. This intervention was associated with an increased risk of ICP compared to that in progesterone-only controls, highlighting the need for clinical vigilance regarding maternal safety. Although there was no significant between-group difference in the incidence of ICP in the sensitivity analysis, this null finding is likely due to the small number of events and limited statistical power. Notably, ICP should remain a safety signal that warrants attention when estrogen supplementation is administered during early pregnancy. These findings indicate that while estradiol supplementation may enhance early reproductive endpoints, its clinical utility remains uncertain due to the absence of ultimate efficacy improvement and emerging safety concerns.

**STUDY FUNDING/COMPETING INTEREST(S):**

The grant support was provided by: the Key R&D Program of Zhejiang (2024C03199), Zhejiang Provincial Medical and Health Technology Plan (2020KY620, 2025KY1176), Zhejiang Provincial Natural Science Foundation of China (LQ24H040002, LHZQN26H280003), Administration of Traditional Chinese Medicine of Zhejiang Province, China (2024ZL740), and Hangzhou Municipal Special Fund for the Development of the Biomedicine and Health Industry (2023WJC327). There are no conflicts of interest to declare.

**TRIAL REGISTRATION NUMBER:**

ChiCTR1900022631.

**TRIAL REGISTRATION DATE:**

19 April 2019.

**DATE OF FIRST PATIENT’S ENROLMENT:**

20 April 2019.

WHAT DOES THIS MEAN FOR PATIENTS? This study looked at whether adding vaginal estrogen to the usual progesterone treatment during the luteal phase (i.e. the time after oocyte retrieval when undergoing IVF or ICSI fertility treatments) could help improve the chances of getting pregnant and having a baby. For people having IVF or ICSI using a GnRH antagonist protocol, doctors sometimes worry that estrogen levels might drop too low after treatment, making it harder for an embryo to attach to the uterus and for a pregnancy to take hold; researchers have therefore tested adding vaginal estrogen alongside usual progesterone. In this study, patients were split into two groups: one received standard progesterone treatment, the other progesterone plus vaginal estrogen. The results showed that estrogen helped more embryos to implant and led to more early positive pregnancy tests (confirmed by ultrasound), but it did not significantly improve the number of pregnancies continuing past the first 3 months or the number of live births. Importantly, the estrogen group had a higher rate of intrahepatic cholestasis of pregnancy, a liver condition that causes itching and may require closer medical monitoring during pregnancy. For patients, this means that adding vaginal estrogen may help initial embryo attachment but does not improve the ultimate goal of having a baby. The possible risk of intrahepatic cholestasis means that vaginal estrogen should be used carefully, and patients should talk through the benefits and risks with their doctors to make a well-informed decision fitting their health and treatment goals.

## Introduction

In recent decades, the prevalence of infertility has been increasing globally, and has been attributed to various factors such as social changes and environmental degradation (Keating *et al.*, 2024; Sakali *et al.*, 2024). Assisted reproductive technology, especially IVF-embryo transfer (IVF-ET), has emerged as a crucial solution. Over the past 40 years, IVF-ET has made remarkable progress. However, the clinical pregnancy rate in reproductive centres worldwide has remained around 50%, with the live birth rates per transfer cycle ranging from 20% to 30% (Kupka *et al.*, 2024).

IVF-ET necessitates ovarian hyperstimulation to obtain an adequate number of oocytes (Gerber *et al.*, 2020). Among the diverse ovarian stimulation protocols, the gonadotropin-releasing hormone antagonist (GnRH antagonist) protocol has become popular due to its advantages such as shorter treatment duration and reduced ovarian hyperstimulation (Si *et al.*, 2024). However, recent research has indicated that GnRH antagonists might affect estrogen levels and endometrial receptivity, resulting in a relatively lower fresh embryo transfer pregnancy rate compared to the GnRH agonist protocol (Rackow *et al.*, 2008; Lambalk *et al.*, 2017). This has led to the proposal of estrogen supplementation during luteal support in the GnRH antagonist protocol.

While the benefits of progesterone supplementation are widely accepted, the role of estrogen supplementation is still under debate in the application of ART. Previous research has yielded inconsistent results. A meta-analysis in 2008 showed that there was no statistically significant difference in the clinical pregnancy rate between the estrogen supplementation group and the progesterone-only group in either the agonist or antagonist protocol (Gelbaya *et al.*, 2008). In contrast, a meta-analysis including of 11 studies indicated that progesterone+estrogen had a higher likelihood of clinical pregnancy than progesterone alone (Zhang *et al.*, 2015). Vaginal administration of micronized estradiol effectively improves endometrial thickness in patients who failed to reach adequate thickness with oral estradiol (Tourgeman *et al.*, 2001). Notably, vaginal estrogen administration confers higher local endometrial estrogen concentrations and a more favourable systemic hormonal profile relative to the oral route. In previous studies of long GnRH agonist protocols, the vaginal administration of estrogen for luteal support was associated with better pregnancy rates compared to oral administration (Fatemi *et al.*, 2006; Elgindy *et al.*, 2010). Existing randomized controlled trials (RCTs) on estrogen supplementation in the GnRH antagonist protocol are scarce and mainly involve oral administration with small sample sizes. While one RCT indicated a potential benefit of oral luteal estradiol supplementation in improving the embryo implantation rate (Kwon *et al.*, 2013), other evidence showed that it did not lead to a significant improvement in either the clinical pregnancy rate or the ongoing pregnancy rate (Ismail Madkour *et al.*, 2016). Currently, there is a notable absence of RCT research investigating vaginal estrogen supplementation in the GnRH antagonist protocol, which our study aims to address.

As the GnRH antagonist protocol is increasingly used in clinical practice, the need to optimize luteal support has become urgent. Investigating the optimal regimen and dosage of estrogen supplementation, with emphasis on the efficacy of vaginal administration, has emerged as a prominent research focus within the domain of reproductive medicine. This study aimed to fill this knowledge gap by conducting an RCT to evaluate the impact of vaginal estrogen supplementation on pregnancy outcomes in the GnRH antagonist protocol, to provide valuable insights for clinicians and policymakers in the reproductive field.

## Materials and methods

### Study design and participants

This randomized single-centre clinical trial aimed to evaluate the impact of luteal estrogen supplementation vaginally in the GnRH antagonist protocol on the pregnancy outcomes of IVF/ICSI cycles, which was conducted from April 2019 to June 2022. The study received approval from the Institutional Review Board of Women’s Hospital, Zhejiang University School of Medicine (No. 20190023) and was registered with the Chinese Clinical Trial Registry (registration number: ChiCTR1900022631). All participants provided their written informed consent before being enrolled in the study, ensuring the ethical and legal compliance of the research. The data were stored in the electronic medical record system. All the investigators ensured the accuracy and integrity of the data, along with the fidelity of the trial to the protocol.

The study involved women undergoing IVF or ICSI using the GnRH antagonist protocol with HMG or FSH for ovarian stimulation. Participants were aged between 20 and 39 years and were on their first or second IVF/ICSI cycle. They had a BMI of less than 28 kg/m^2^ and maintained regular menstrual cycles every 24–35 days. Patients in this cycle were scheduled for fresh cleavage-stage (day-3) embryo transfer.

Exclusion criteria included couples with contraindications to IVF/ICSI, diminished ovarian reserve (DOR, defined as a basal FSH level greater than 12 IU/ml and a total antral follicle count of fewer than 3), scheduling for preimplantation genetic testing (PGT), severe male factor infertility requiring testicular sperm extraction, unavailability of oocytes, polycystic ovary syndrome (PCOS), severe endometriosis (stages III or IV), hydrosalpinges, an hCG day progesterone level exceeding 1.5 ng/ml, endometrial thickness of less than 8 mm, frozen-thawed cycles, or high risk of ovarian hyperstimulation syndrome (OHSS) risk (hCG-day E_2_ >20 000 nmol/l or oocytes retrieved >20).

### Randomization

Prior to the commencement of ovarian stimulation, the recruited women were randomly allocated into two groups at a 1:1 ratio using software PASS 15 (NCSS, LLC., Kaysville, UT, USA): the Progesterone group and Progesterone + estradiol group. This randomization list was rendered by an independent data manager (F.L.). It was not feasible to implement blinding for either the women or the reproductive medicine physicians. However, during the data analysis phase, the statisticians remained unaware of the group allocation until the completion of the statistical analysis.

### Sample size

Based on the assumption of a 43% ongoing pregnancy rate in the treatment group and a 30% ongoing pregnancy rate in the control group (Zhang *et al.*, 2018; Qiao *et al.*, 2021; Xu *et al.*, 2024), which is regarded as clinically significant, a sample size of 212 in each group would afford 80% power at a 5% significance level to detect a difference of 13% (Fatemi *et al.*, 2006; Elgindy *et al.*, 2010). Considering an anticipated loss rate of 10%, the initial prestudy sample size for each group was determined to be 236.

### Luteal phase support

#### P group

In the luteal phase, patients received a daily vaginal administration of 90 mg progesterone (Crinone 8% vaginal gel, Merck Serono, Feltham, UK) and an oral intake of 10 mg dydrogesterone (Duphaston, Abbott, Amsterdam, the Netherlands) twice per day. This treatment regimen was initiated on the second day following oocyte retrieval. Serum β-hCG testing was carried out 10 days after the embryo transfer. If pregnancy was successfully confirmed, the treatment was continued for a duration of 10 weeks.

#### E ± P group

The treatment protocol for this group was identical to that of the Progesterone group, with the addition of a nightly vaginal insertion of 2 mg estradiol (Femoston 2/10 red tablet, Abbott, Weesp, the Netherlands). The administration of estradiol commenced on the day after oocyte retrieval and continued until the completion of 8 weeks of gestation, which corresponded to the timepoint for a scan to confirm the viability of the foetal heart, provided that pregnancy was achieved.

### Outcomes

The principal outcome was the attainment of an ongoing pregnancy subsequent to a single cycle of fresh IVF/ICSI. An ongoing pregnancy was operationalized as the visualization of a foetal heartbeat via ultrasonography beyond 10-week gestation.

The secondary outcomes encompassed the implantation rate, clinical pregnancy rate, miscarriage rate, ectopic pregnancy rate, and live birth rate. The implantation rate was quantified as the ratio of the number of gestational sacs visualized on ultrasound to the number of embryos transferred. Clinical pregnancy, miscarriage, ectopic pregnancy, and live birth were defined as reported previously (Shi *et al.*, 2018).

OHSS is an iatrogenic complication of ovulation induction in ART, characterized by ovarian enlargement and increased vascular permeability, leading to massive extravasation of body fluids and accumulation in the abdominal cavity, thoracic cavity, and other sites (Practice Committee of the American Society for Reproductive Medicine, 2006). Preterm labour refers to delivery at a gestational age of ≥28 weeks and <37 weeks (Subgroup of Obstetrics and Gynecology, 2024). Hypertensive disorders of pregnancy (HDP) denote a constellation of conditions defined by gestational-onset or exacerbated hypertension, often accompanied by proteinuria, end-organ dysfunction, or maternal-foetal complications. This diagnostic spectrum encompasses chronic hypertension, gestational hypertension, preeclampsia, and eclampsia (Magee *et al.*, 2022). Gestational diabetes mellitus (GDM) was diagnosed in pregnant women without pre-existing diabetes when any single plasma glucose value met or exceeded the 75-g oral glucose tolerance test thresholds at 24–28 weeks of gestation: fasting ≥5.1 mmol/l, 1-h ≥10.0 mmol/l, or 2-h ≥8.5 mmol/l (International Association of Diabetes and Pregnancy Study Groups Consensus Panel *et al.*, 2010). Intrahepatic cholestasis of pregnancy (ICP) is defined as the occurrence of unexplained palm and sole pruritus and abnormal liver function during gestation, with symptoms resolving after delivery in the absence of primary skin or hepatobiliary lesions (Horgan *et al.*, 2023).

### Statistical analysis

For the presentation of categorical baseline data, absolute numbers and percentages were utilized. Normally distributed continuous variables were expressed as mean accompanied by standard deviation (SD), while non-normally distributed continuous variables were summarized as median along with the 25th to 75th percentile. To compare normally distributed continuous outcomes, independent *t*-tests were employed. In the case of non-normally distributed continuous outcomes, Mann–Whitney *U*-tests were carried out for comparison. For the comparison of dichotomous variables between groups, chi-square tests were applied. Relative risk (RR) with 95% CI was also calculated. A sensitivity analysis was performed using multivariable logistic regression, with adjustment for age, BMI, AMH, number of top-quality embryos, and number of transferred embryos. In all analyses, a two-tailed *P*-value less than 0.05 was regarded as statistically significant. All statistical analyses were executed using SPSS software (version 16.0, SPSS Inc., Chicago, IL, USA).

## Results

### Baseline characteristics

**Figure 1.**
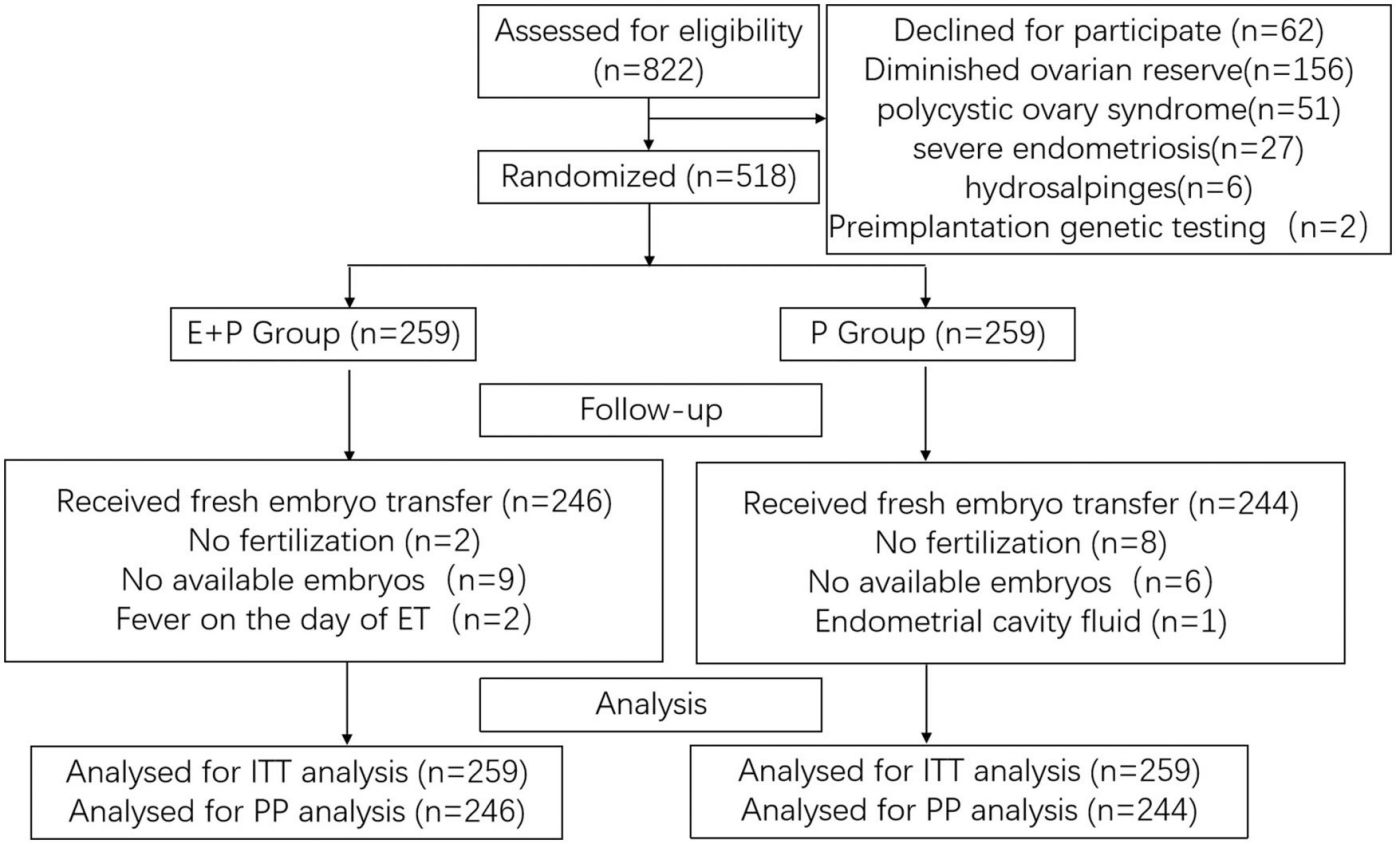
**Flowchart of the study**. P group, progesterone-only luteal support; E+P group, progesterone plus estradiol for luteal support; ET, embryo transfer; ITT, intention-to-treat; PP, per protocol.

Between April 2019 and June 2022, a total of 822 infertile women were initially screened for the present study. Subsequently, after the application of exclusion criteria, which encompassed 62 couples who declined participation, 156 cases presenting with DOR, 51 diagnoses of PCOS, 27 cases of severe endometriosis, 6 diagnoses of hydrosalpinges, and 2 couples necessitating PGT, a total of 518 participants were ultimately enrolled in the study. A recruited women underwent randomization after trigger, with 259 assigned to progesterone-only luteal support (P group) and 259 to progesterone plus estradiol (E + P group). Figure 1 illustrates the study flowchart.

The baseline characteristics of the E + P group showed no significant differences compared to the P group (Table 1). None of those women were lost to follow-up. A total of 13 women dropped out of the E + P group while 15 from the P group dropped out. In the E + P group, two patients experienced fertilization failure, nine had no viable embryos for transfer, and two cancelled their embryo transfer cycles due to fever on the day of ET. Similarly, in the P group, eight patients had fertilization failure, six lacked viable embryos, and one cancelled the fresh ET cycle because of endometrial cavity fluid.

**Table 1.** Baseline characteristics and statistical analysis of the study cohort.

	ITT		PP	
	E + P (n = 259)	P (n = 259)	E + P (n = 246)	P (n = 244)
**Age (years)**	30.61 ± 3.75	30.67 ± 3.70	30.58 ± 3.78	30.68 ± 3.64
**Duration of infertility (years)**	3.00 (1.50–4.00)	3.00 (2.00–5.00)	3.00 (1.38–4.00)	3.00 (2.00–4.00)
**Duration of cycle (days)**	30.00 (28.00–34.00)	30.00 (28.00–33.00)	30.00 (28.00–34.00)	30.00 (28.00–33.00)
**BMI (kg/m^2^)**	21.76 ± 2.66	21.74 ± 2.42	21.76 ± 2.67	21.74 ± 2.43
**FSH (IU/l)**	6.67 ± 1.54	6.68 ± 1.51	6.63 ± 1.49	6.72 ± 1.53
**E_2_ (pmol/l)**	108.65 ± 45.68	110.88 ± 60.00	108.44 ± 45.39	109.43 ± 58.52
**AMH (ng/ml)**	3.21 (2.19–4.68)	3.39 (2.24–4.60)	3.20 (2.19–4.66)	3.40 (2.33–4.70)
**AFC**	13.69 ± 4.27	13.19 ± 4.10	13.65 ± 4.30	13.24 ± 4.15
**Infertility**				
Primary	126/259 (48.65%)	144/259 (55.60%)	121/246 (49.19%)	133/244 (54.51%)
Secondary	133/259 (51.35%)	115/259 (44.40%)	125/246 (50.81%)	111/244 (45.49%)

Data are reported as mean (SD), median (range), and n/total n (%) as applicable. AFC, antral follicle count; AMH, anti-Müllerian hormone; E_2_, estradiol; ITT, intention-to-treat; PP, per protocol.

### IVF/ICSI treatment characteristics

The treatment characteristics of IVF/ICSI cycles are presented in Table 2. No significant differences were observed between the two groups regarding the duration of ovarian stimulation, total gonadotropin (Gn) dosage, number of retrieved oocytes, fertilized oocytes, or top-quality embryos.

**Table 2.** Demographic and IVF/ICSI treatment parameters of study participants.

	ITT			PP		
	E + P (n = 259)	P (n = 259)	*P*-values	E + P (n = 246)	P (n = 244)	*P*-values
**Ovarian stimulation duration**	9.55 ± 1.41	9.63 ± 1.59	0.559	9.56 ± 1.42	9.60 ± 1.55	0.735
**Units of Gn (IU)**	1950.00 (1575.00–2250.00)	2025.00 (1650.00–2325.00)	0.100	1975.00 (1575.00–2250.00)	2025.00 (1725.00–2375.00)	0.099
**E_2_ on the hCG day (pmol/l)**	8128.00 (6116.00–10 168.00)	8308.00 (6812.00–9657.00)	0.434	8443.00 (6018.00–10 210.25)	8396.00 (6918.00–9575.00)	0.471
**P on the hCG day (nmol/l)**	2.06 (1.52–2.90)	2.17 (1.63–2.85)	0.387	2.08 (1.54–2.90)	2.20 (1.64–2.90)	0.396
**No. of retrieved oocytes**	9.00 (7.00–12.00)	10.00 (8.00–12.00)	0.209	10.00 (7.00–12.00)	10.00 (8.00–13.00)	0.062
**Fertilization method**						
IVF	183 (70.66%)	175 (67.57%)	0.804	177 (71.95%)	166 (68.03%)	0.557
ICSI	67 (25.87%)	76 (29.34%)		61 (24.80%)	71 (29.10%)	
HALF ICSI	9 (3.47%)	8 (3.09%)		8 (3.25%)	7 (2.87%)	
**No. of fertilized oocytes**	5.00 (3.00–7.00)	5.00 (3.00–8.00)	0.125	5.00 (3.00–7.00)	5.00 (4.00–8.00)	0.082
**Top-quality embryos**	2.00 (1.00–4.00)	2.00 (1.00–4.00)	0.473	2.00 (1.00–4.00)	2.00 (2.00–4.00)	0.31
**E_2_ on day of ET (pmol/l)**	5960.00 (4675.00–7408.00)	5090.00 (3852.50–6557.00)	<0.0001	5986.00 (4652.25–7393.75)	5085.00 (3850.25–6596.50)	<0.0001
**P on day of ET (nmol/l)**	242.00 (175.00–325.00)	255.00(199.45–331.00)	0.048	241.68 (173.03–325.00)	255.05 (200.75–334.21)	0.019
**E_2_ at Day 10 post-ET**	3808.00(2298.50–5826.50)	919.35 (103.50–3446.00)	<0.0001	3642.00 (2283.75–5244.00)	654.60 (93.30–3294.00)	<0.0001
**Embryo transferred**						
SET	28/259 (10.81%)	36/259 (13.90%)	0.350	28/246 (11.38%)	36/244 (14.75%)	0.268
DET	218/259 (84.17%)	208/259 (80.31%)		218/246 (88.62%)	208/244 (85.25%)	

Data are reported as mean (SD), median (range), and n/total n (%) as applicable. DET, double-embryo transfer; ET, embryo transfer; E_2_, estradiol; Gn, gonadotropins; ITT, intention-to-treat; P, progesterone; PP, per protocol; SET, single-embryo transfer.

Serum E_2_ and P levels on the day of hCG administration were comparable between the groups. However, following E + P treatment, a significant elevation in E_2_ levels was detected on the embryo transfer (ET) day in both the intention-to-treat (ITT) and per-protocol (PP) analyses. Furthermore, E_2_ concentrations measured 10-day post-ET demonstrated a marked increase in the E + P group compared to the P groups.

### Primary outcomes

The addition of estradiol did not result in a statistically significant improvement in the ongoing pregnancy rate (Table 3). According to the ITT analysis, the ongoing pregnancy rate was higher in the E + P group (46.33%, 120/259) compared to the P group (39.00%, 101/259), although this difference did not reach statistical significance (RR 1.19, 95% CI: 0.97–1.45; *P *= 0.091). In the PP analysis, consistent results were observed with ongoing pregnancy rates of 48.78% (120/246) in the E + P group versus 41.39% (101/244) in the P group (RR 1.18, 95% CI: 0.97–1.44; *P *= 0.100).

**Table 3.** Pregnancy outcomes following fresh embryo transfer in the GnRH antagonist protocol.

	ITT						PP					
	E + P (n = 259)	P (n = 259)	*P-values*	RR (95% CI)	Adjusted *P*-value	Adjusted OR^†^ (95% CI)	E + P (n = 246)	P (n = 244)	*P-values*	RR (95% CI)	Adjusted *P*-value	Adjusted OR^†^ (95% CI)
**Primary outcomes**												
Ongoing pregnancy rate	120/259 (46.33%)	101/259 (39.00%)	0.091	1.19 (0.97–1.45)	0.114	1.35 (0.93–1.96)	120/246 (48.78%)	101/244 (41.39%)	0.100	1.18 (0.97–1.44)	0.111	1.35 (0.93–1.96)
**Secondary outcomes**												
Implantation rate	184/464 (39.66%)	149/452 (32.96%)	0.035	1.20 (1.01–1.43)	–	–	184/464 (39.66%)	149/452 (32.96%)	0.035	1.20 (1.01–1.43)	–	–
Clinical-pregnancy rate	138/259 (53.28%)	114/259 (44.02%)	0.035	1.21 (1.01–1.44)	0.046	1.45 (1.01–2.10)	138/246 (56.10%)	114/244 (46.72%)	0.038	1.20 (1.01–1.43)	0.043	1.46 (1.01–2.11)
Ectopic pregnancy rate	3/259 (1.16%)	2/259 (0.77%)	0.653	1.50 (0.25–8.90)	0.611	1.60 (0.26–9.75)	3/246 (1.22%)	2/244 (0.82%)	0.660	1.49 (0.25–8.83)	0.610	1.60 (0.26–9.76)
Miscarriage rate	20/259 (7.72%)	14/259 (5.41%)	0.287	1.43 (0.74–2.77)	0.289	1.47 (0.72–2.98)	20/246 (8.13%)	14/244 (5.74%)	0.297	1.42 (0.73–2.74)	0.272	1.49 (0.73–3.04)
Live birth rate	116/259 (44.79%)	98/259 (37.84%)	0.108	1.18 (0.96–1.46)	0.139	1.32 (0.91–1.92)	116/246 (47.15%)	98/244 (40.16%)	0.119	1.17 (0.96–1.44)	0.137	1.33 (0.92–1.92)
**Complications**												
OHSS	2/259 (0.77%)	1/259 (0.39%)	0.563	2.00 (0.18–21.92)	0.662	1.72 (0.15–19.60)	2/246 (0.81%)	1/244 (0.41%)	0.567	2.00 (0.18–21.92)	0.662	1.72 (0.15–19.60)
Premature birth	27/116 (23.28%)	15/98 (15.31%)	0.144	1.52 (0.86–2.69)	0.158	1.67 (0.82–3.39)	27/116 (23.28%)	15/98 (15.31%)	0.144	1.52 (0.86–2.69)	0.158	1.67 (0.82–3.39)
HDP	6/116 (5.17%)	3/98 (3.06%)	0.443	1.69 (0.43–6.58)	0.438	1.77 (0.42–7.54)	6/116 (5.17%)	3/98 (3.06%)	0.443	1.69 (0.43–6.58)	0.438	1.77 (0.42–7.54)
GDM	19/116 (16.38%)	15/98 (15.31%)	0.831	1.07 (0.57–1.99)	0.801	1.10 (0.52–2.34)	19/116 (16.38%)	15/98 (15.31%)	0.831	1.07 (0.57–1.99)	0.801	1.10 (0.52–2.34)
ICP	6/116 (5.17%)	0/98 (0%)	0.032*	–	0.996	–	6/116 (5.17%)	0/98 (0%)	0.032*	–	0.996	–

Data are reported as n/total n (%). GDM, gestational diabetes mellitus; HDP, hypertensive pregnancy disorder; ICP, intrahepatic cholestasis of pregnancy; ITT, intention-to-treat; PP, per protocol; OHSS, ovarian hyperstimulation syndrome; RR, relative risk; OR, odds ratio.

* Fisher’s exact test.

† Data were adjusted for age, BMI, AMH, number of top-quality embryo, and number of transfer embryo using multivariable logistic regression.

### Secondary outcomes

The ITT analysis revealed significantly higher clinical pregnancy rates in the E + P group (53.28%, 138/259) compared to the P group (44.02%, 114/259), with a relative risk of 1.21 (95% CI: 1.01–1.44; *P *= 0.035) (Table 3). This finding was corroborated by the PP analysis, demonstrating clinical pregnancy rates of 56.10% (138/246) in the E + P group versus 46.72% (114/244) in the P group (RR: 1.20; 95% CI: 1.01–1.43; *P *= 0.038).

Implantation rates, in both the ITT and PP analyses, also favoured the E + P group (39.66% vs 32.96%; RR 1.20, 95% CI: 1.01–1.43; *P* = 0.035). However, no significant differences in live birth rates were found between the groups.

### Complications

OHSS occurred in three cases (2 in E + P group versus 1 in P group) (Table 3). Among pregnant women, the incidence rates of preterm birth, HDP, and GDM showed no significant differences between groups. However, ICP was significantly more frequent in the E + P group (6/116, 5.17%) compared to the P group (0/98, 0%) (*P* = 0.032). After a sensitivity analysis with adjustment for age, BMI, AMH, the number of top-quality embryos, and the number of transferred embryos, the incidence of ICP did not differ significantly between groups (Adjusted *P* = 0.996).

## Discussion

In 2020, the ESHRE guidelines recommended the GnRH antagonist protocol as the top choice for ovulation induction in patients with normal ovarian response (The ESHRE Guideline Group on Ovarian Stimulation *et al.*, 2020). This is primarily attributed to its streamlined treatment process, which not only simplifies the overall course of treatment but also effectively shortens the treatment duration. Additionally, it is characterized by a notably low incidence of OHSS, further enhancing its desirability and safety profile in clinical applications. Nevertheless, previous research has demonstrated that the pregnancy outcomes of fresh embryo transfer using the GnRH-ant protocol are less than satisfactory compared with GnRH agonist protocol (Xu *et al.*, 2020). A meta-analysis has shown that the ongoing pregnancy rate in women with normal ovarian response using the GnRH antagonist protocol is significantly lower than that in those using the GnRH agonist protocol, with the rate being 23.8% for the former and 27.4% for the latter (Lambalk *et al.*, 2017). Endometrial receptivity, rather than embryo quality, is potentially the pivotal factor that underlies this particular observation (Zhang *et al.*, 2021; Xu *et al.*, 2022). The improvement of pregnancy outcomes for these patients has emerged as a significant challenge within the realm of current clinical practice.

In the GnRH antagonist protocol, estrogen supplementation to strengthen luteal support may be necessary. This may be because the antagonist can directly bind to GnRH receptors in endometrial cells, thus affecting endometrial receptivity; on the other hand, the antagonist may cause early luteolysis, leading to aggravated luteal insufficiency. The current randomized single-centre clinical trial was designed to explore the impact of vaginal estrogen supplementation during the luteal phase in the GnRH antagonist protocol on pregnancy outcomes in IVF/ICSI cycles. The results obtained provide valuable insights into the ongoing debate regarding the role of estrogen in luteal support.

During the fresh embryo transfer cycle under the antagonist protocol, the vaginal administration of estrogen in the embryo implantation period and the early luteal support stage has the potential to augment both the embryo implantation rate and the early pregnancy rate. One possible explanation for this phenomenon lies in the intricate hormonal interplay during early pregnancy. Estrogen acts as a key regulator, influencing multiple cellular processes within the endometrium. It has been demonstrated that estrogen can modulate the expression of adhesion molecules on endometrial cells, such as increased expression of integrins, facilitating a more favourable environment for embryo attachment (Chen *et al.*, 2016; Dias Da Silva *et al.*, 2024). Moreover, estrogen also plays a part in angiogenesis within the endometrium, which is essential for the developing embryo, and estrogen-induced angiogenesis ensures the delivery of necessary nutrients and oxygen (Moon *et al.*, 2022). Adequate estrogen supplementation is conducive to promoting endometrial metaplasia and elevating the endometrial receptivity (Yang *et al.*, 2021). In the present study, although not statistically significant, the rates of miscarriage and ectopic pregnancy were numerically higher in the E + P group. There were previous findings indicating that estradiol supplementation during intrauterine insemination cycles, while increasing endometrial lining thickness, was also associated with significantly higher odds of miscarriage (Zhang *et al.*, 2024). This might have indirectly resulted in no statistically significant difference in the ongoing pregnancy rate and live birth rate between the E + P group and the P group in the final analysis. Therefore, we believe that the addition of vaginal estrogen during the peri-transplantation period and early pregnancy may have improved endometrial receptivity and increased the implantation rate, but had no significant effect on pregnancy maintenance, resulting in no significant difference between the two groups in terms of ongoing pregnancy and live birth.

Our follow-up data revealed that there was no significant difference in the rates of ectopic pregnancy and miscarriage between the two groups. This finding is particularly noteworthy as previous research had indicated a considerably higher ectopic pregnancy rate in fresh transfer cycles of IVF/ICSI compared to frozen transfer cycles, potentially attributed to elevated estrogen levels (Zargar *et al.*, 2021). However, in our current investigation, despite the estrogen-supplemented group exhibiting substantially higher estrogen levels on the day of transfer than the P group, there was no disparity in the ectopic pregnancy rate. This outcome provides evidence to validate the safety of estrogen supplementation. However, it suggests that other factors, which warrant further exploration, might be overriding the potential adverse impact of elevated estrogen on ectopic pregnancy occurrence.

During the follow-up in the late pregnancy and perinatal period, it was observed that the incidence of ICP in patients receiving estrogen-progesterone supplementation after antagonist-induced ovulation was higher than those administered progesterone alone. However, no significant between-group difference in ICP incidence was identified in the sensitivity analysis, which might be attributed to the limited number of events and insufficient statistical power. Nevertheless, ICP remains a clinically relevant safety concern and warrants close surveillance as a potential complication associated with estrogen supplementation during pregnancy. The onset of ICP occurred between 20 and 37 weeks of gestation. Of the six ICP cases diagnosed, severity was classified based on serum total bile acid levels: one case was severe (TBA ≥40 μmol/l), and five were mild (TBA 10–39 μmol/l). All affected patients were managed with ursodeoxycholic acid, alone or in combination with S-adenosylmethionine, with a good clinical response. With the exception of one vaginal delivery due to precipitate labour, all other deliveries in the ICP group were performed via caesarean section. All neonates from these pregnancies exhibited favourable outcomes with no adverse events attributed to ICP. This clinical observation raises important safety considerations regarding the potential association between exogenous estrogen administration and ICP risk in ART cycles. The pathophysiological basis for this association may be multifactorial. While the precise aetiology of ICP remains to be fully elucidated, accumulating evidence implicates estrogen homeostasis as a key modulator in this disease pathogenesis (Hobson *et al.*, 2022). The physiological estrogen surge during pregnancy directly modulates bile acid homeostasis, potentially compromising hepatobiliary transport mechanisms and predisposing to ICP pathogenesis. Estrogen has been shown to downregulate the protein levels of the bile salt export pump (BSEP) and multidrug resistance-associated protein 2 (key transporters in bile acid homeostasis) and furthermore, 17β-estradiol suppresses BSEP expression in human hepatocytes, thereby reducing bile acid secretion (Song *et al.*, 2014; Xiao *et al.*, 2021). Notably, research has emphasized that pregnant women undergoing IVF/ICSI treatment have a substantially higher prevalence of ICP than those who conceived naturally (11.4% vs 4.3%) (Zhu *et al.*, 2016). However, the body of existing evidence is mired in contention. In contrast to the inference regarding estrogen supplementation in the IVF/ICSI cycle exacerbating ICP, certain studies had posited that women with ICP actually exhibited lower levels of estrogen and dehydroepiandrosterone sulphate (Leslie *et al.*, 2000; Xiao *et al.*, 2021).

However, it is important to note that this study has its limitations. The lack of blinding for participants and clinicians introduces potential performance and detection bias. Specifically, knowledge of treatment allocation might have influenced patient-reported outcomes and adherence, as well as clinician’s decisions regarding adjuvant medications or the interpretation of symptoms and ultrasound findings. While the primary and secondary outcomes are objective, the clinical management decisions preceding it could have been unconsciously influenced by the knowledge of treatment allocation, introducing a potential source of performance bias. Even so, despite our efforts to further mitigate this issue by blinding statisticians during data analysis, the absence of double-blinding remains a significant concern. Second, the sample size, while calculated to achieve adequate power, may still be insufficient to detect subtle differences in pregnancy outcomes, particularly in secondary endpoints. Third, the generalizability of our findings is limited by the single-centre design and the homogeneous East Asian population, as luteal physiology and estrogen metabolism may vary across ethnicities. Moreover, the current study focused exclusively on D3 cleavage-stage embryo transfer; the effect of estrogen supplementation following fresh blastocyst transfer remains unclear and warrants further investigation through randomized controlled trials. Finally, outcomes related to ICP may not be fully representative due to regional variations in incidence. Future studies with a larger sample size, multicentre design, and more rigorous blinding methods are warranted to further elucidate the role of estrogen supplementation on the pregnancy outcomes in the GnRH antagonist protocol.

In conclusion, this study has provided valuable insights into the role of vaginal estrogen supplementation as luteal phase support in GnRH antagonist cycles for IVF/ICSI patients. While an increase in the implantation rate and clinical pregnancy rate was observed with the addition of estradiol, it was important to note that there were no significant differences in the ongoing pregnancy rate or live birth rate between the E + P and P groups. This suggests that although estradiol supplementation may have a positive impact on the initial stages of embryo implantation, other factors might be at play in determining the ultimate success of a pregnancy. Future research should focus on identifying these factors and further exploring the optimal dosage and administration route of estradiol to potentially enhance the overall pregnancy outcomes. Additionally, larger and multicentred studies are warranted to validate our findings and provide more conclusive evidence regarding the true value of luteal estradiol supplementation in this context.

## Data Availability

The data underlying this article are available in the article. Data sets from clinical research will not be shared and no other documents will be available. Access criteria for data sharing are not applicable. Individual participant data will not be available.
